# Changes in coronal plane alignment of the knee classification do not significantly influence outcomes after restricted kinematic alignment total knee arthroplasty

**DOI:** 10.1002/jeo2.70602

**Published:** 2025-12-17

**Authors:** Simon Messe, Guillaume Mesnard, Hannes Vermue, Enrico Festa, Elvire Servien, Anthony Viste, Cécile Batailler, Sébastien Lustig

**Affiliations:** ^1^ Orthopaedics Surgery and Sports Medicine Department FIFA Medical Centre of Excellence, Croix‐Rousse Hospital Lyon University Hospital Lyon France; ^2^ Department of Orthopaedic Surgery Ghent University Hospital Gent Belgium; ^3^ Department of Public Health, Orthopedic Unit “Federico II” University Naples Italy; ^4^ LIBM – EA 7424, Interuniversity Laboratory of Biology of Mobility Claude Bernard Lyon 1 University Lyon France; ^5^ Hospices Civils de Lyon, Hôpital Lyon Sud, Chirurgie Orthopédique et Traumatologique Pierre Bénite France; ^6^ University Lyon, Claude Bernard Lyon 1 University, Gustave Eiffel University Lyon France; ^7^ Univ Lyon, Claude Bernard Lyon 1 University Lyon France

**Keywords:** alignment strategy, CPAK classification, functional outcome, knee biomechanics, restricted kinematic alignment, total knee arthroplasty

## Abstract

**Purpose:**

The coronal plane alignment of the knee (CPAK) classification helps understand knee alignment variability, guiding personalised total knee arthroplasty (TKA) strategies. However, evidence regarding the impact of postoperative CPAK classification changes on patient‐reported functional outcomes after restricted kinematic alignment (rKA) TKA remains limited. This study aimed to investigate whether CPAK classification changes after TKA with rKA influence functional outcomes, measured by the International Knee Society score (IKS).

**Methods:**

A retrospective cohort study included 464 patients who underwent primary TKA with a posterior stabilised implant (KNEO^®^) between January 2020 and May 2024. The inclusion criteria were primary or secondary osteoarthritis with complete radiographic and clinical follow‐up at 2 years; patients with incomplete data or intraoperative complications requiring implant change were excluded. Pre‐ and postoperative CPAK classifications were compared, and functional outcomes were assessed using IKS knee and function scores at 2 years of follow‐up. Radiographic assessment was performed on standing long‐leg radiographs by a single experienced observer.

**Results:**

A significant redistribution of CPAK classifications was observed postoperatively (*p* < 0.001), with 22.2% of CPAK I changing to CPAK II and 19.1% changing to CPAK V. In total, 33.3% of all knees retained their initial CPAK classification. No significant differences were observed in the postoperative IKS Knee (85.2 ± 12.3 vs. 83.9 ± 11.8; *p* = 0.62) or IKS Function scores (78.5 ± 13.1 vs. 76.9 ± 12.7; *p* = 0.54). Given the small sample sizes within certain CPAK subtype transitions, subgroup analyses were not feasible.

**Conclusions:**

Changes in CPAK classification following rKA‐TKA were common but did not significantly influence functional outcomes at 2 years. These findings suggest that CPAK phenotype transition alone may not be a reliable predictor of clinical success, although larger studies are needed to explore subtype‐specific effects.

**Level of Evidence:**

Level IV.

## Graphical abstract

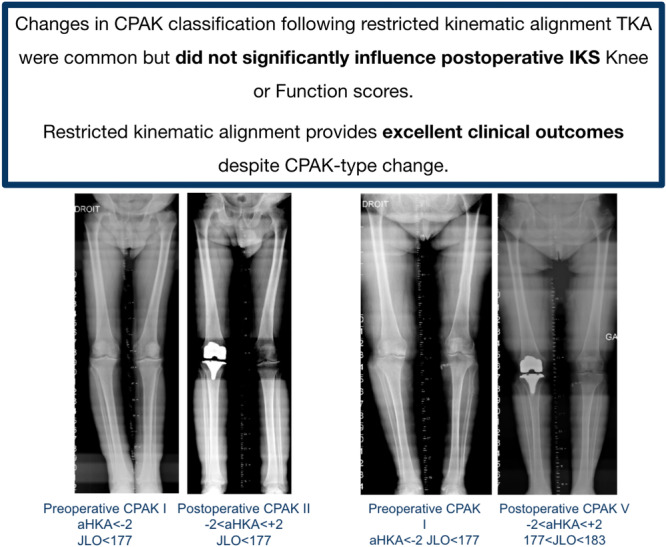

**Abbreviations**
aHKA: arithmetic hip‐knee‐ankle angle
ASA: American Society of Anesthesiologists
BMI: body mass index
CPAK: coronal plane alignment of the knee
IKS: International Knee Society (score)
JLO: joint line obliquity
KA: kinematic alignment
LDFA: lateral distal femoral angle
MA: mechanical alignment
MCL: medial collateral ligament
MPTA: medial proximal tibial angle
PACS: picture archiving and communication system
PROMs: patient‐reported outcome measures
PSA: patient‐specific alignment
rKA: restricted kinematic alignment
TKA: total knee arthroplasty

## INTRODUCTION

Total knee arthroplasty (TKA) remains one of the most commonly performed surgical procedures for treating knee osteoarthritis [26]. However, despite generally favourable outcomes, a significant proportion of patients remain dissatisfied after surgery, with revision rates often linked to alignment issues and implant wear [13]. Traditionally, TKA has been based on mechanical alignment (MA), aiming to restore a neutral lower limb axis [19], and to ensure implant longevity. Although MA has shown satisfactory clinical results in many studies, a notable proportion of patients still experience poor long‐term functional outcomes, suggesting that this standardised approach may not address the specific needs of all patients [31].

To overcome these challenges, new realignment strategies have been proposed, including kinematic alignment (KA) and restricted kinematic alignment (rKA) [30]. KA, which aims to restore the pre‐arthritic anatomy of the knee, has shown promising results in terms of patient satisfaction and function, although not strictly superior over the MA philosophy [6]. As such, the question remains as to the best way to address the anatomical and biomechanical variations in the knee, particularly regarding joint line obliquity (JLO) and coronal alignment [6, 14].

The coronal plane alignment of the knee (CPAK) classification, a modern approach to classifying knee phenotypes, provides a better understanding of coronal alignment variability and allows for a more personalised surgical strategy [25, 26]. Recent large‐scale analyses using artificial intelligence have further demonstrated sex‐specific variability in CPAK and functional phenotypes, highlighting the complexity of constitutional knee alignment [15]. By combining the angles of the lateral distal femoral angle (LDFA) and medial proximal tibial angle (MPTA), this classification offers a more comprehensive view of constitutional alignment and JLO [28]. Although some studies have suggested that restoring preoperative anatomical parameters may improve function and reduce pain, other reports have shown inconsistent or contradictory results depending on the alignment technique used [25]. Moreover, recent evidence has shown that CPAK classification cannot be used to determine segmental coronal extra‐articular deformities, further questioning its applicability as a comprehensive alignment tool [23].

Previous large‐scale studies, such as Kraus et al. [22], have investigated the impact of CPAK type changes on outcomes, but subgroup analyses are limited by sample size in smaller cohorts. Furthermore, existing evidence on the effect of CPAK transitions in rKA‐TKA remains sparse.

Therefore, the aim of this study was to determine whether changes in CPAK classification from preoperative to postoperative status influence functional outcomes after rKA‐TKA. We hypothesised that patients who maintained their preoperative CPAK type would achieve better postoperative functional outcomes, as measured by the International Knee Society (IKS) score, compared with those who transitioned to a different CPAK type.

## MATERIALS AND METHODS

### Study design

This was a retrospective study that included all patients who underwent primary TKA using the Posterior Stabilised KNEO^®^ (Groupe Lépine) implant at two tertiary referral centres specialised in primary and revision knee arthroplasty between January 2020 and May 2024. This study was approved by the national ethical committee (approval number ID‐RCB 2020‐A00414‐35) and conducted in accordance with the 1964 Helsinki declaration and its later amendments.

### Setting and participants

**Figure 1:**
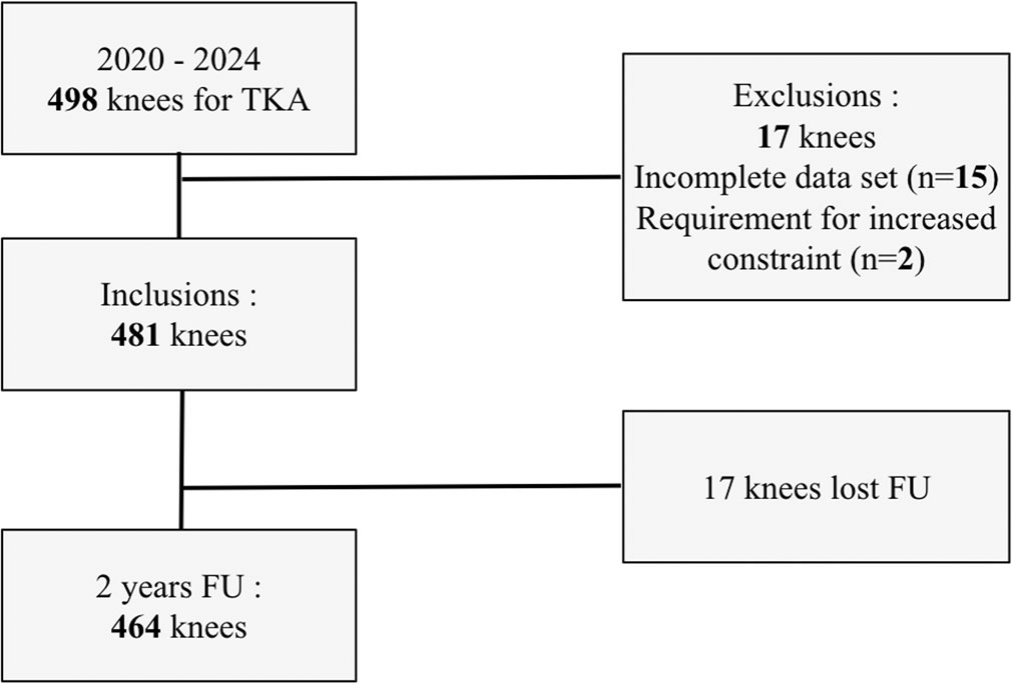
Patient selection flowchart. TKA, total knee arthroplasty.

The study included patients requiring TKA for diagnoses of primary osteoarthritis, inflammatory arthritis, avascular necrosis or posttraumatic arthritis. Posttraumatic cases were included only if complete fracture union and restoration of limb alignment were confirmed on preoperative full‐length radiographs. Patients with incomplete data were excluded from the analysis (Figure 1). In addition, two patients were excluded because intraoperative events required a change in the prosthetic constraint level. Specifically, both patients sustained a medial collateral ligament rupture during surgery, which necessitated conversion from a posterior‐stabilised implant to a more constrained prosthetic design. In total, 11 surgeons performed the procedures: four trained senior consultants with over 10 years of TKA experience, and seven assistant surgeons.

The patient demographics are summarised in Table 1. A total of 498 patients were eligible for review from June 2020 to May 2024. The study included 464 patients with a follow‐up of 2 years.

**Table 1:** Baseline demographics.

Variable	Value	Range
Mean age, years (SD)	71.7 (8.1)	35−91
Mean BMI, kg/m² (SD)	29.0 (5.2)	15−55
Sex, W % (*n*)	64% (297)	
Laterality, L % (*n*)	43% (200)	
Mean surgical time, minutes (SD)	73.5 (20.2)	
ASA 1% (*n*)	6.9 (32)	
ASA 2% (*n*)	64.9 (301)	
ASA 3% (*n*)	27.8 (129)	
ASA 4% (*n*)	0.4 (2)	
Primary osteoarthritis % (*n*)	88.7 (412)	
Posttraumatic osteoarthritis % (*n*)	7.7 (36)	
Postnecrotic osteoarthritis % (*n*)	1.4 (6)	
Postrheumatic osteoarthritis % (*n*)	2.2 (10)	

Abbreviations: ASA, American Society of Anesthesiologists; BMI, body mass index; L, Left; SD, standard deviation; W, Women.

### Surgical technique

All patients underwent a standardised enhanced recovery protocol, including spinal anaesthesia, early mobilisation, appropriate pain management and thromboprophylaxis as per national guidelines [10]. The procedures were performed through a subvastus, medial parapatellar or lateral surgical approach, using a manual measured resection technique within the framework of rKA. Intramedullary referencing was utilised for both the femur and tibia. All TKAs were implanted according to the rKA philosophy, as previously described by Venditolli [3]. In this study, only the rKA technique was applied; functional alignment was not used. The algorithm employs a ‘safe range’ approach, where the femoral and tibial bone cuts are modified to be within ±5 degrees of the mechanical axis, and the hip‐knee‐ankle (HKA) angle is maintained within ± 3 degrees of neutral alignment. The femoral and tibial component was always cemented. Patellar resurfacing was performed selectively based on the degree of patellar osteoarthritis observed during the surgery.

### Data collection

Preoperatively, the following parameters were collected through screening of the patients electronical records: age, gender, body mass index (BMI), American Society of Anesthesiologists (ASA) score, side of surgery, osteoarthritis aetiology, IKS (both knee and function) and CPAK types. IKS scores were prospectively collected during routine clinical follow‐up and retrospectively extracted from the institutional database for analysis. The IKS [7] score is widely used to assess the clinical outcomes of knee surgeries, including TKA. This scoring system includes two distinct components: the knee score and the function score. The IKS Knee score evaluates clinical aspects such as pain, range of motion and knee stability, while the Function score measures the patient′s ability to perform activities of daily living and engage in physical activities.

**Figure 2:**
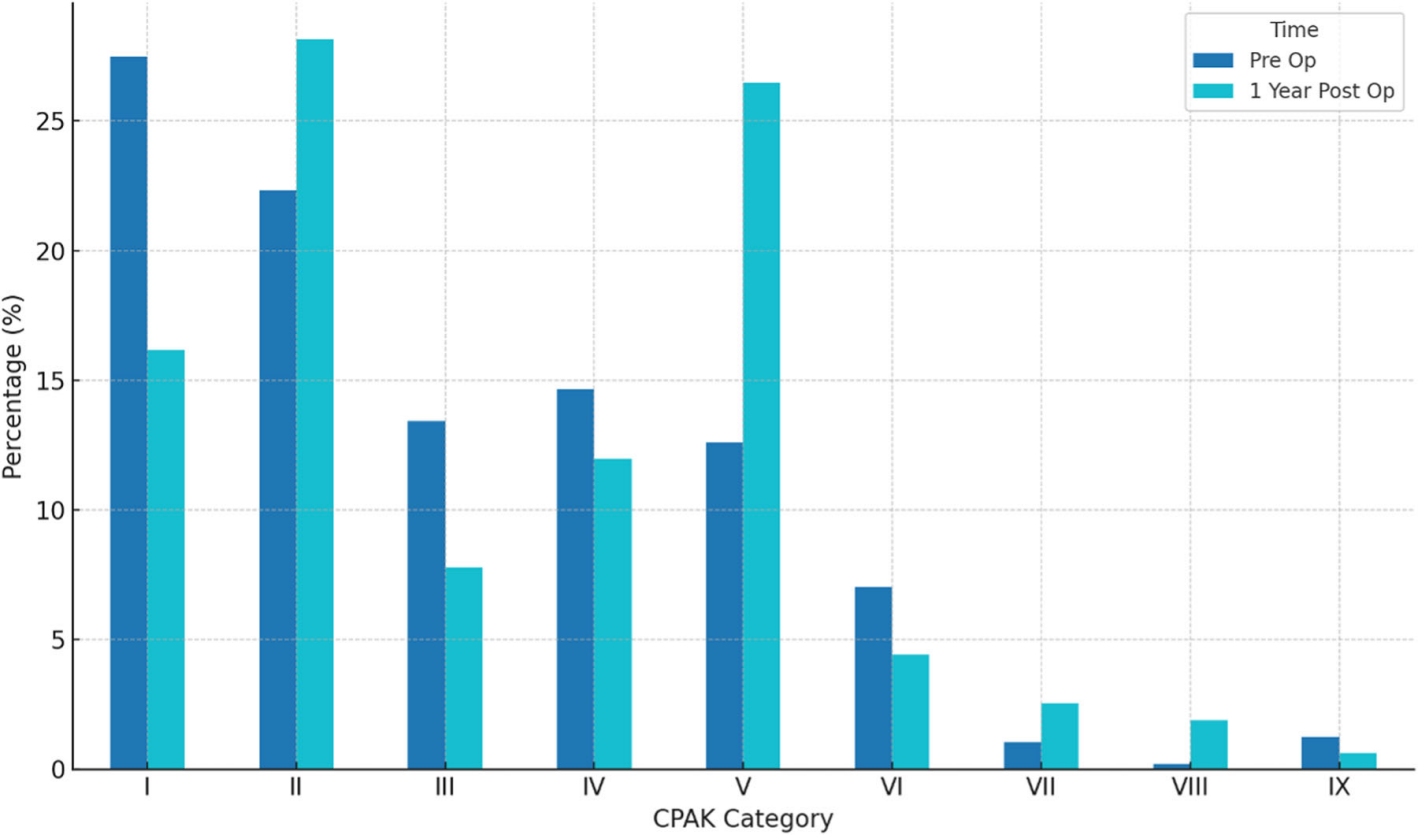
Distribution of CPAK classification types before and 1 year after total knee arthroplasty (TKA). Bar chart illustrating the percentage of patients in each of the nine coronal plane alignment of the knee (CPAK) categories preoperatively (dark blue) and postoperatively (light blue). While preoperative distribution was primarily centred in types I, a significant postoperative shift is observed, with a marked increase in type II and V representation. These changes suggest a redistribution of mechanical alignment profiles following restricted kinematic alignment TKA. The vertical axis represents the proportion of patients in each category, and the horizontal axis lists the CPAK types I–IX.

Postoperative data collected included patient satisfaction and IKS scores at 2 years postoperatively, as well as CPAK types 1 year after the procedure. Mean follow‐up was 35.2 ± 8 months (range: 24–56). The distribution of patients across the different CPAK categories in the pre‐operative and postoperative phases (at 1 year) is shown Table 2 and illustrated in Figure 2.

**Table 2:** Evolution of CPAK classification before and after total knee arthroplasty.

CPAK category	Pre op % (*n*)	1 year post op % (*n*)
I	27.2 (126)	12.3 (57)
II	22.2 (103)	26.9 (125)
III	13.1 (61)	4.7 (22)
IV	14.9 (69)	15.1 (70)
V	12.7 (59)	26.9 (125)
VI	7.3 (34)	6 (28)
VII	1.1 (5)	4.7 (22)
VIII	0.2 (1)	2.4 (11)
IX	1.3 (6)	0.9 (4)

Abbreviation: CPAK, coronal plane alignment of the knee.

### Radiological measurements

All radiological measurements were performed with PACS 11.4 (Carestream Health Inc.) using standardised standing long‐leg radiographs. All radiographs were assessed on standardised standing long‐leg images (PACS 11.4, Carestream). Two fellowship‐trained orthopaedic surgeons independently measured LDFA and MPTA on all cases while blinded to each other′s measurements and clinical outcomes. Arithmetic hip‐knee‐ankle angle (aHKA) (MPTA − LDFA) and JLO (LDFA + MPTA) were derived per rater. Interobserver reliability was good for LDFA (intraclass correlation coefficient [ICC] = 0.88; 95% CI, 0.85–0.91), MPTA (ICC = 0.86; 95% CI, 0.83–0.89), aHKA (ICC = 0.87; 95% CI, 0.84–0.90) and JLO (ICC = 0.85; 95% CI, 0.82–0.88). Intraobserver reliability was excellent for both raters (ICC range, 0.91–0.95). All ICCs used a two‐way random‐effects, absolute‐agreement, single‐measure model (ICC[2,1]). The limbs were categorised into CPAK types using the approach described by MacDessi et al. [24]. An evaluator, who was not blinded to the study results, determined the aHKA angle, measuring the MPTA and LDFA and applying the formula: aHKA = MPTA–LDFA. The JLO was calculated as the sum of the MPTA and LDFA. The CPAK classification describes the direction of the JLO as ‘apex distal’, ‘neutral’ or ‘apex proximal’, based on the location of the apex of the angle formed by the intersection of the joint lines of both knees at the midline relative to a horizontal joint as outlined by MacDessi et al. [24]. The boundaries for a neutral arithmetic HKA angle are 0° ± 2°, with a varus aHKA less than −2° and a valgus aHKA greater than +2°. The boundaries for a neutral JLO are 180° ± 3°, with an apex distal JLO less than 177° and an apex proximal JLO greater than 183° [24]. Stable CPAK was defined as patients remaining in the same CPAK class pre and postoperatively, whereas the transitioned CPAK group defined patients who changed CPAK class from pre to postoperatively.

### Statistical analysis

Continuous variables were assessed for normality using Shapiro–Wilk test. Normally distributed continuous variables are presented as mean ± standard deviation. Parametric data were analysed using Student's *t*‐tests, whereas nonparametric data were evaluated using Mann–Whitney *U*‐tests.

To compare IKS knee and function scores across CPAK transition groups, Mann–Whitney *U*‐tests were applied. Similarly, preoperative HKA values between patients with and without posttraumatic osteoarthritis were compared using a two‐sample *t*‐test.

To compare preoperative and postoperative CPAK classifications, a contingency table was constructed. Bowker′s test was applied to evaluate the symmetry of CPAK distributions before and after surgery. Pairwise comparisons of CPAK transitions were analysed using McNemar′s test to identify specific statistically significant changes.

A post‐hoc power analysis was conducted for the comparison of postoperative IKS Knee scores between stable and transitioned CPAK groups. Given the small effect size (Cohen′s *d* = 0.11), the statistical power was only 8.3%, confirming that the study was underpowered for subtype‐specific analyses.

The significance threshold was set at an α of 0.05. All statistical analyses were performed using SPSS (version 26.0, IBM) and R (version 4.0.5, R Foundation).

## RESULTS

### Contingency table analysis

**Figure 3:**
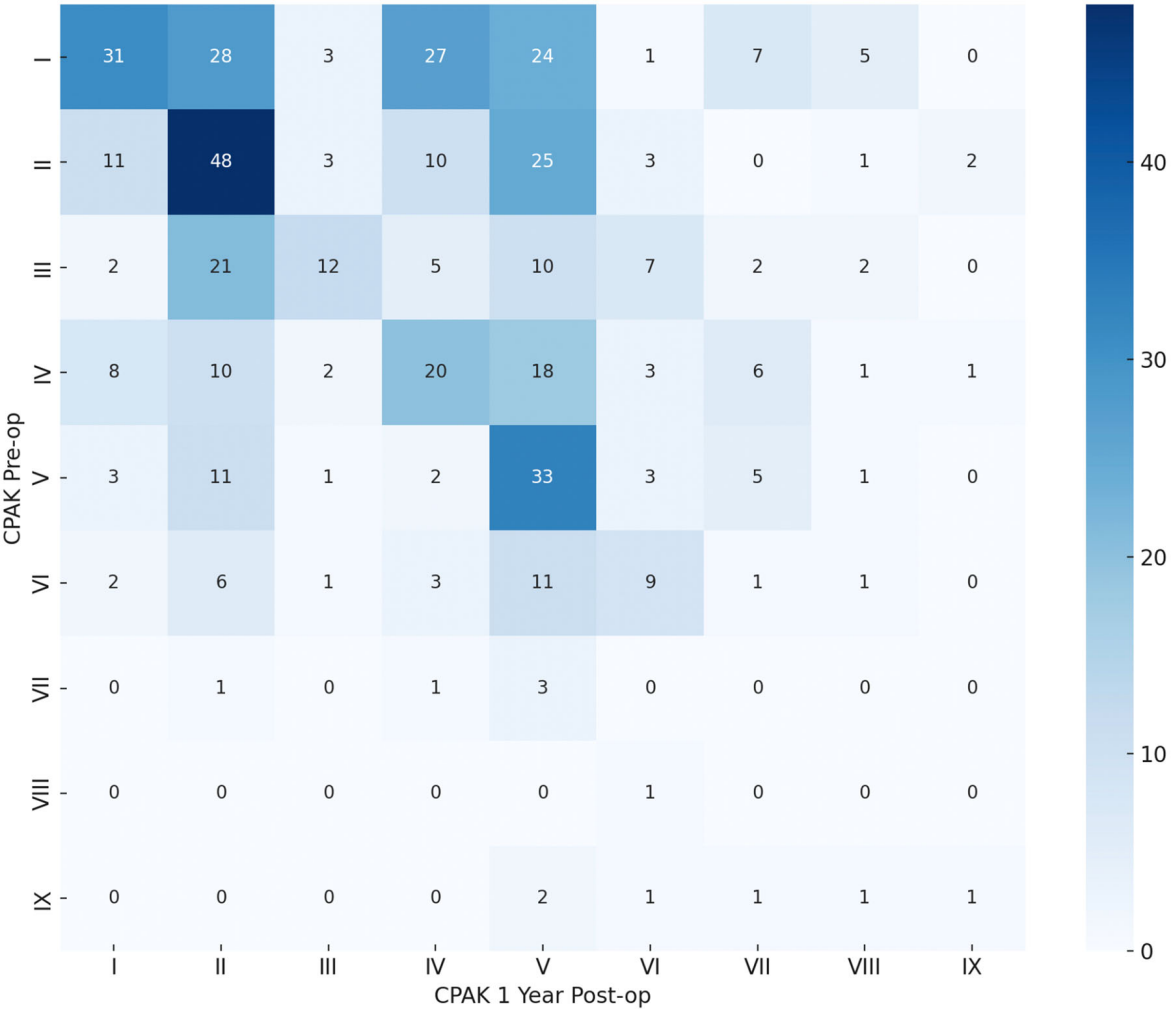
Contingency table of patient distribution across CPAK classifications before and after surgery. This contingency table illustrates the distribution of patients according to their CPAK classification before and 1 year after total knee arthroplasty. Each cell represents the number of patients who transitioned from a preoperative CPAK group (vertical axis) to a postoperative CPAK group (horizontal axis). The darker cells indicate a higher concentration of patients, highlighting the most frequent transition patterns. CPAK, coronal plane alignment of the knee.

The contingency table (Figure 3) provides a detailed comparison of preoperative and postoperative CPAK classifications.

The overall distribution of CPAK classifications changed significantly from pre to postoperative status (Bowker′s test, *p* < 0.001). Specifically, 22.2% of CPAK I patients changed to CPAK II and 19.1% changed to CPAK V. Additionally, significant changes were also noted from CPAK II to CPAK V (*p* < 0.001), CPAK IV to CPAK V (*p* < 0.001) and CPAK III to CPAK II (*p* < 0.001). Overall, 33.3% of knees retained their initial CPAK classification.

**Figure 4:**
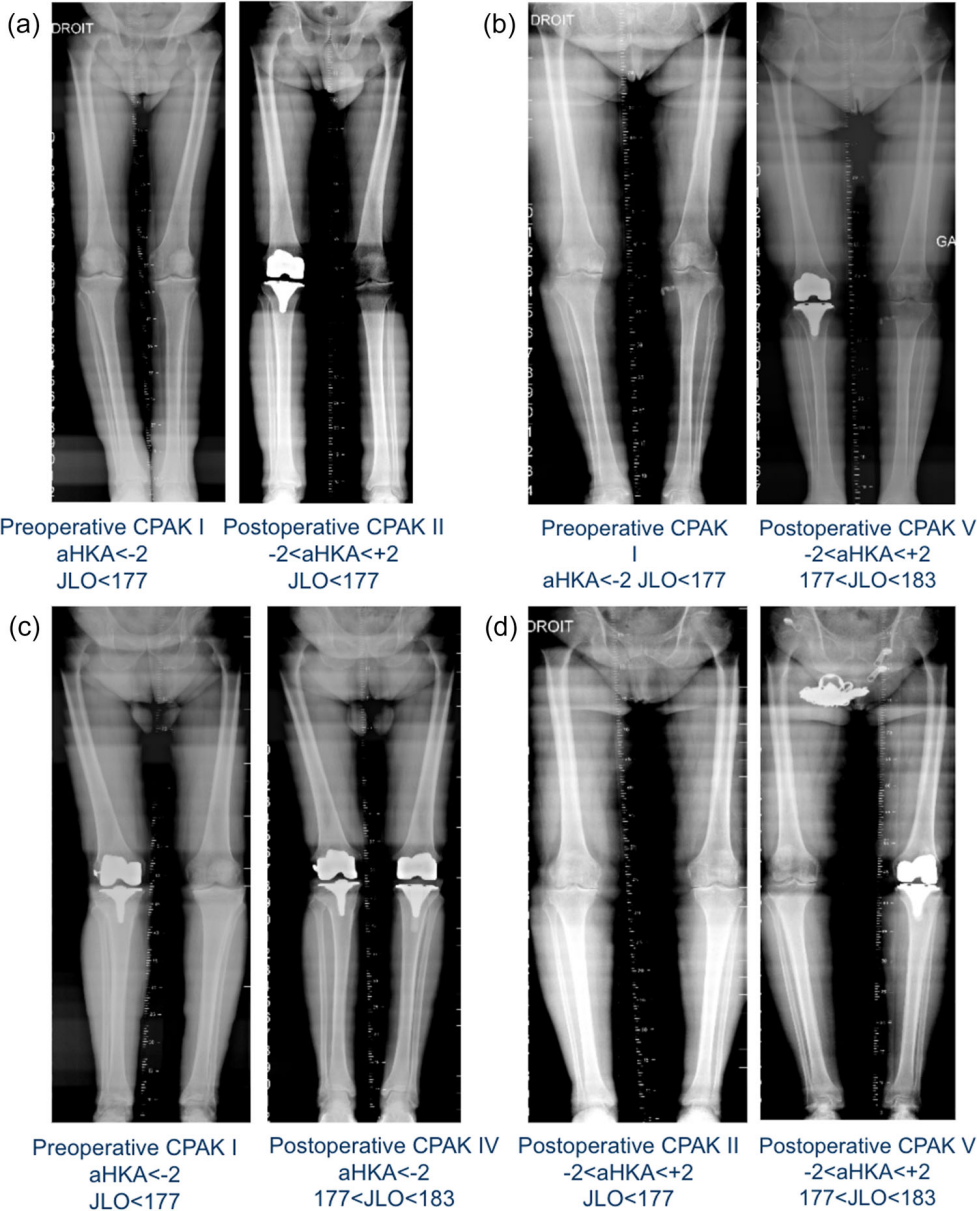
Representative pre‐ and postoperative long‐leg radiographs illustrating the most frequent CPAK transitions observed in this study: (a) CPAK I → II, (b) CPAK I → V, (c) CPAK I → IV and (d) CPAK II → V. aHKA, arithmetic hip‐knee‐ankle angle; CPAK, coronal plane alignment of the knee; JEO, joint line obliquity.

Representative pre‐ and postoperative long‐leg radiographs illustrating the most frequent CPAK transitions (I → II, I → V, I → IV and II → V) are shown in Figure 4.

### HKA analysis in post‐traumatic osteoarthritis

The analysis of HKA preoperative values between patients with post‐traumatic osteoarthritis and those without showed no significant difference. Patients with posttraumatic osteoarthritis had a mean HKA value of 177.7° (standard deviation [SD] = 7.47), while patients without posttraumatic osteoarthritis had a mean HKA value of 176.06° (SD = 6.83). There was no significant difference between the two groups (*p* = 0.16). Impact of CPAK change on IKS score evolution.

IKS knee and function scores were compared across CPAK transition groups (Table 3). Among the 464 patients, 3.4% (*n* = 16) underwent a valgus to varus transition, 2.2% (*n* = 10) underwent a varus to valgus transition and 94.4% (*n* = 438) experienced other transitions. The mean postoperative IKS knee scores for these groups were 84.4 (SD 14.0), 89.0 (SD 8.1) and 85.2 (SD 11.5), respectively.

**Table 3:** Comparison of IKS scores across CPAK transition groups.

Group		IKS knee (mean ± SD)	*p* value	IKS function (mean ± SD)	*p* value
Valgus → varus % (*n*)	3.4% (16)	84.4 ± 14	0.93	80.3 ± 20.9	0.82
Varus → valgus % (*n*)	2.2% (10)	89 ± 8.1	0.43	79 ± 16.8	0.57
Other transitions % (*n*)	94.4% (438)	85.2 ± 11.5	—	81.3 ± 15.3	—

*Note*: Summary of the number and percentage of patients by CPAK transition group (valgus to varus, varus to valgus and other transitions), with corresponding mean IKS knee and function scores.

Abbreviations: CPAK, coronal plane alignment of the knee; IKS, International Knee Society; SD, standard deviation.

Corresponding IKS function scores were 80.3 (SD 20.9), 79.0 (SD 16.8) and 81.3 (SD 15.3). No statistically significant differences were observed in IKS knee scores (*p* = 0.93 for valgus to varus vs. others; *p* = 0.43 for varus to valgus vs. others) or IKS function scores (*p* = 0.82 and *p* = 0.57, respectively).

The mean preoperative IKS function score was 59.2 (SD = 16.0), and the mean preoperative IKS knee score was 60.1 (SD = 12.2). Postoperatively, there was a significant improvement, with the mean IKS function score rising to 81.5 (SD = 15.4) (*p* < 0.001) and the IKS knee score reaching 85.1 (SD = 11.4) (*p* < 0.001).

**Figure 5:**
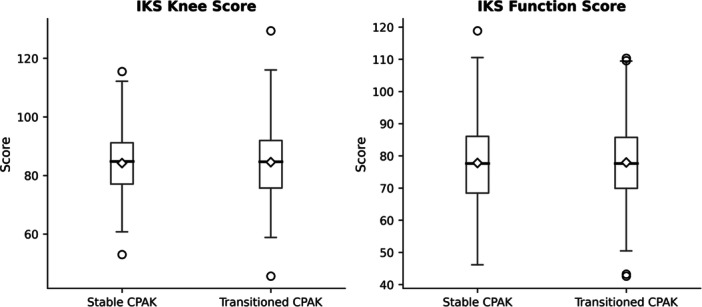
Box plots comparing postoperative IKS knee and function scores between patients with stable and transitioned CPAK classifications (*n* = 464). CPAK, coronal plane alignment of the knee; IKS, International Knee Society.

As illustrated in Figure 5, postoperative IKS Knee and Function scores were comparable between patients who maintained their CPAK classification and those who transitioned to a different type.

Mean IKS Knee scores were 85.2 (SD = 12.3) in the stable CPAK group and 83.9 (SD = 11.8) in the transitioned group (*p* = 0.62), while mean IKS Function scores were 78.5 (SD = 13.1) and 76.9 (SD = 12.7), respectively (*p* = 0.54). A post hoc power analysis comparing postoperative IKS Knee scores between stable and transitioned CPAK groups yielded a statistical power of 8.3%, indicating that the study was underpowered to detect small intergroup differences.

## DISCUSSION

### CPAK classification and outcomes

This study aimed to assess the impact of changes in CPAK classification on functional outcomes following TKA performed with rKA [5]. Our findings indicate that alterations in CPAK classification from preoperative to postoperative status did not significantly influence functional outcomes, as measured by the IKS score. These findings contribute to the ongoing discussion on the relationship between coronal plane knee phenotype and postoperative function.

Several studies have emphasised the importance of alignment in predicting functional outcomes after TKA [23, 32, 33]. Pangaud et al. [27] reported that restoring the preoperative CPAK phenotype was associated with improved functional scores, particularly in the ‘activities of daily living’ and ‘quality of life’ subscales of the KOOS. Kraus et al. [22] also found that maintaining the native CPAK phenotype postoperatively did not improve functional scores at 24 months. Among 2427 TKA patients, only 11.5% retained their preoperative CPAK classification, with significant shifts in alignment patterns. No overall association was found between CPAK alignment and outcomes, except for CPAK I, which showed higher postoperative pain than CPAK III (*p* = 0.027).

In the present series, although many patients transitioned in the CPAK classification from pre‐ to postoperatively, no significant differences in IKS scores were observed between stable and transitioned CPAK groups. This aligns with the observations of Jagota et al. [17] and Rodriguez et al. [29], who noted that CPAK classification does not fully account for the three‐dimensional variability of knee anatomy, limiting its predictive value. In fact, Jenny et al. [18] demonstrated that CPAK does not correlate with functional phenotype classification, reinforcing the notion that CPAK alone is insufficient to predict clinical outcomes. Additionally, Şahbat et al. [30] found that CPAK classification correctly identified the knee joint apex in only 42.7% of cases, suggesting that it may not fully capture knee morphology variations. Similarly, Huber et al. [15] demonstrated sex‐specific variability in CPAK and functional phenotypes in a large cohort of over 8000 osteoarthritic knees, reinforcing the limitations of CPAK in capturing the full spectrum of alignment variability. These findings highlight the need to refine CPAK classification for better alignment‐based surgical planning. Given the small sample size in certain CPAK subtype transitions, zero were underpowered and no definitive conclusions can be drawn regarding their individual impact on outcomes. In contrast, Araki et al. [2] proposed a modified version of CPAK classification based on restricted kinematic alignment (Rb‐CPAK), which may offer a more refined understanding of knee biomechanics. Future studies should evaluate whether this classification system provides a better correlation with functional outcomes, potentially offering a more predictive model for postoperative knee function.

### Alignment and outcomes beyond CPAK

It should be underlined that the absence of an effect of CPAK transitions on outcomes does not mean that alignment itself is irrelevant.

Several reports support the role of alignment. Graichen et al. [12] highlighted the effectiveness of patient‐specific alignment (PSA) techniques in restoring alignment in varus and neutral knees while normalising valgus knees and severe varus deformities. These findings suggest that rather than strictly adhering to CPAK classification, PSA may offer superior functional outcomes, particularly in cases of significant deformities. Our study, conducted with rKA, supports the notion that while CPAK classification provides valuable insights, transitions in CPAK classification do not necessarily translate into meaningful functional changes [1, 18]. This reinforces the need for individualised surgical strategies that account for multiple biomechanical factors beyond coronal alignment alone [9]. Kobayashi et al. [20] further demonstrated that rKA better restores joint line orientation compared to MA, with 72% of rKA patients achieving a neutral JLO, compared to only 27% in MA. Despite this, no significant functional differences were observed after 3 years, reinforcing our findings. Ettinger et al. [8] directly compared rKA to MA using a medial‐pivot prosthesis, which resulted in better functional scores at 1 year postoperatively and in varus CPAK classes at 2 years for the rKA cohort. Corban et al. [4] reported that rKA offers a more personalised and functionally beneficial alignment strategy for patients with varus phenotypes, compared to the traditional MA approach.

Our analysis also found that neither posttraumatic osteoarthritis nor CPAK transition patterns were associated with significant differences in preoperative alignment or postoperative IKS scores. While Konishi et al. reported that changes in varus/valgus alignment and postoperative JLO negatively impact patient‐reported outcomes after TKA [21], our findings suggest that CPAK shifts—although reflecting mechanical correction—do not necessarily translate into improved clinical recovery. This aligns with the results of Al‐Abbasi et al., who found that changes in coronal plane alignment do not significantly influence patient‐reported outcomes or implant survivorship following TKA [1], reinforcing the multifactorial nature of functional outcomes in this setting.

Our findings, along with those of Franceschetti et al. [11], emphasise that while alignment influences outcomes, postoperative rehabilitation and pain management are likely to be more critical in determining functional recovery. Itou et al. [16] demonstrated that excessive correction of the HKA angle during TKA leads to increased lateral laxity postoperatively. However, they found no significant differences in patient‐reported outcome measures (PROMs), reinforcing the idea that while alignment corrections may impact joint stability, they do not necessarily correlate with improved functional outcomes when measured using traditional scoring systems like IKS.

The retrospective design imposes inherent limitations. The follow‐up period was restricted to 2 years, although it has been reported that functional results are not likely to change substantially beyond this period [33]. The analysis focused primarily on coronal alignment, whereas sagittal alignment and soft tissue balance were not assessed, despite their potential impact on functional outcomes. Moreover, the use of the IKS score, although widely adopted in the literature, may not fully capture all dimensions of knee function.

## CONCLUSION

In this cohort, changes in CPAK classification, including varus–valgus transitions, were not associated with significant differences in functional outcomes following rKA‐TKA. Although substantial shifts in CPAK types were observed postoperatively, no significant differences in IKS scores were detected, and the limited sample size for certain CPAK subtype transitions precludes definitive conclusions. These findings suggest that correction of coronal alignment alone may not be sufficient to determine clinical recovery.

## AUTHOR CONTRIBUTIONS

**Simon Messe**: Study design; data collection; statistical analysis; literature review and manuscript writing. **Guillaume Mesnard**: Study design; literature review and manuscript editing. **Hannes Vermue**: Literature review and manuscript editing. **Enrico Festa**: Literature review and manuscript editing. **Anthony Viste**: Study design; supervision; literature review and manuscript editing. **Cécile Batailler**: Study design; supervision; literature review and manuscript editing. **Cécile Batailler**: Study design; supervision; literature review and manuscript editing. **Sébastien Lustig**: Study design; supervision; literature review and manuscript editing.

## CONFLICT OF INTEREST STATEMENT

Anthony Viste: Consultancy for Stryker and Depuy Synthes. Associate editor for Surgical and Radiologic Anatomy. Cécile Batailler: Consultancy for Groupe Lepine, Smith and Nephew and Stryker. Sébastien Lustig: Royalties from Stryker, Smith and Nephew and consultancy for Heraeus, Depuy Synthes and Serf. Institutional research support from Groupe Lepine and Amplitude. Editorial Board for Journal of Bone and Joint Surgery (Am). The remaining authors declare no conflicts of interest.

## ETHICS STATEMENT

This study was approved by the national ethical committee (approval number [ID‐RCB 2020‐A00414‐35]). Written informed consent was not required for this retrospective study, as all data were anonymised and collected from existing medical records in accordance with institutional and national regulations.

## Data Availability

The data that support the findings of this study are available from the corresponding author upon reasonable request.
